# Supplementary material for: A dual role for the N-terminal domain of the IL-3 receptor in cell signalling
Source: Nat Commun. 2018 Jan 26;9:386. doi: 10.1038/s41467-017-02633-7 (PMC5785977; doi:10.1038/s41467-017-02633-7)
Supplement: Supplementary file 1 — Supplementary Information [file 41467_2017_2633_MOESM1_ESM.pdf]

SUPPLEMENTARY INFORMATION

Supplementary Figures

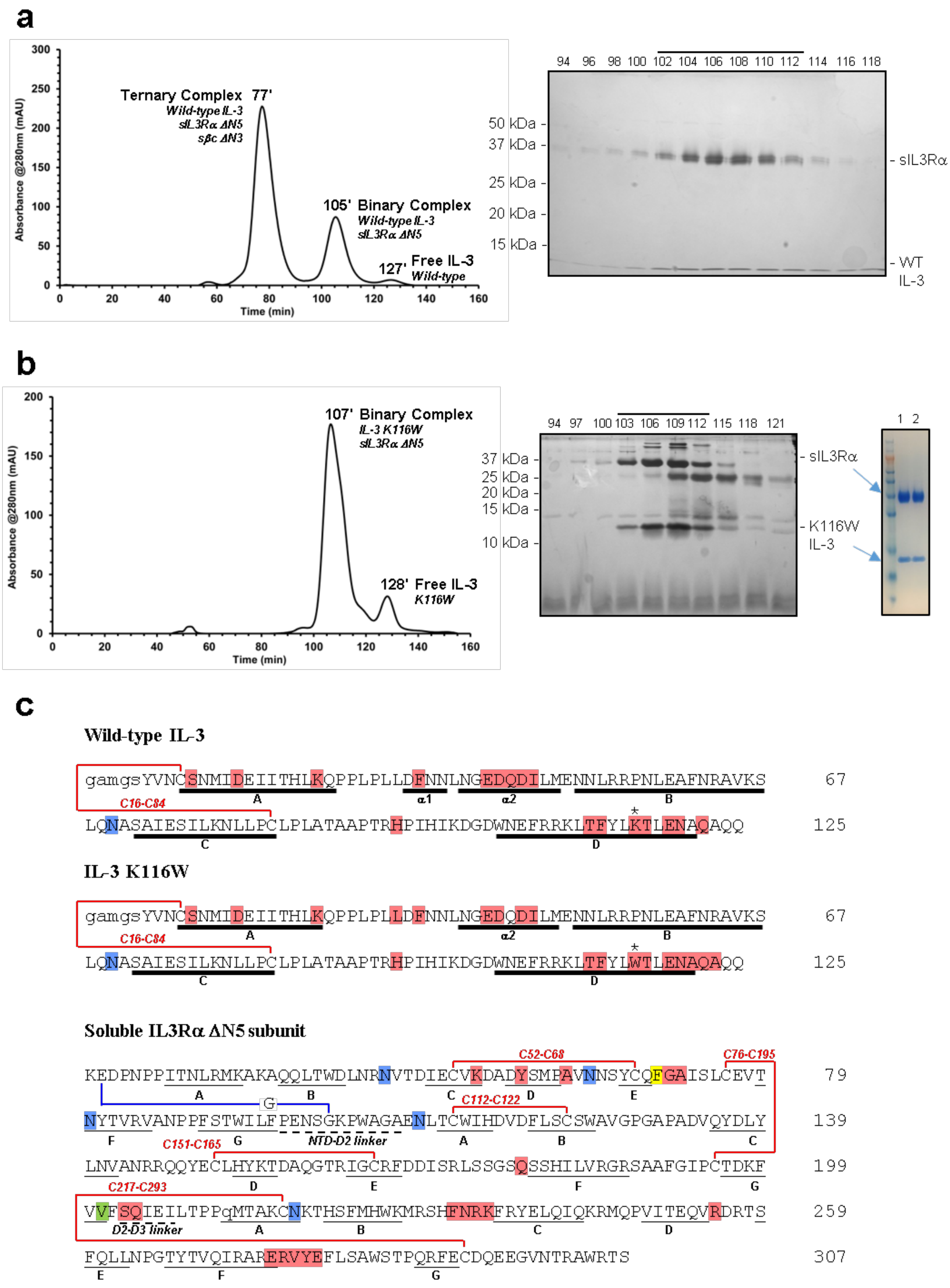

Supplementary Fig. 1. Expression and purification of wild-type IL-3 and IL-3 K116W binary complexes and sequence of components. **a** The wild-type IL-3:sIL3Rα ΔN5 binary complex was purified from a mixture of ternary complex and free IL-3 by size exclusion chromatography. Eluate fractions from the peak eluting at 105 minutes were analysed by Coomassie stained SDS-PAGE and shown to contain the wild-type IL-3:sIL3Rα ΔN5 binary complex (sIL3Rα, WT IL-3). Fractions spanning 102 - 112 minutes were pooled for crystallisation studies. (**b**) The IL-3 K116W:sIL3Rα ΔN5 binary complex was purified from excess IL-3 K116W by size exclusion chromatography. A minor protein was observed eluting at 109 - 118 minutes by SDS-PAGE (25 kDa). Fractions spanning 103 - 112 minutes were pooled, buffer exchanged, concentrated and filtered for crystallisation studies. Protein used for crystallography from two different purifications is shown in right-hand side panel. (**c**) Annotated amino acid sequence of wild-type IL-3 (upper), IL-3 K116W (middle) and sIL3Rα ΔN5 (lower) highlighting the major contacts between IL-3 and sIL3Rα. The sequence of truncated IL-3 (118 residues) shows the position of α helices (A-D, α1 and α2 bold underline), the cysteines linked through a disulfide bond (C-C), the asparagine residue modified by the addition of glycan moieties *in vivo* (blue highlight), the variant K116 or W116 residue (*) and the IL3Rα contact residues (red highlight). The sequence of sIL3Rα ΔN5 (307 residues) shows the position of β-strands (A-G) in each domain, the cysteines linked through disulfide bonds (C-C), the asparagine residues potentially modified by the addition of glycan moieties (blue highlight) and the interdomain linker regions (dashed underline). Mutation of the potential N-linked glycosylation site at N212 is indicated with q212. The common IL-3 contact residues are shown (red highlight) and differentiated for those specific for wild-type IL-3 (green highlight) or IL-3 K116W (yellow highlight). Deletion of the N-terminal domain (NTD) occurs in the SP2 isoform of IL3Rα and fuses E21 to G91 with the inclusion of an additional glycine (denoted as blue line joining E21 and G91).

a

b

c

d

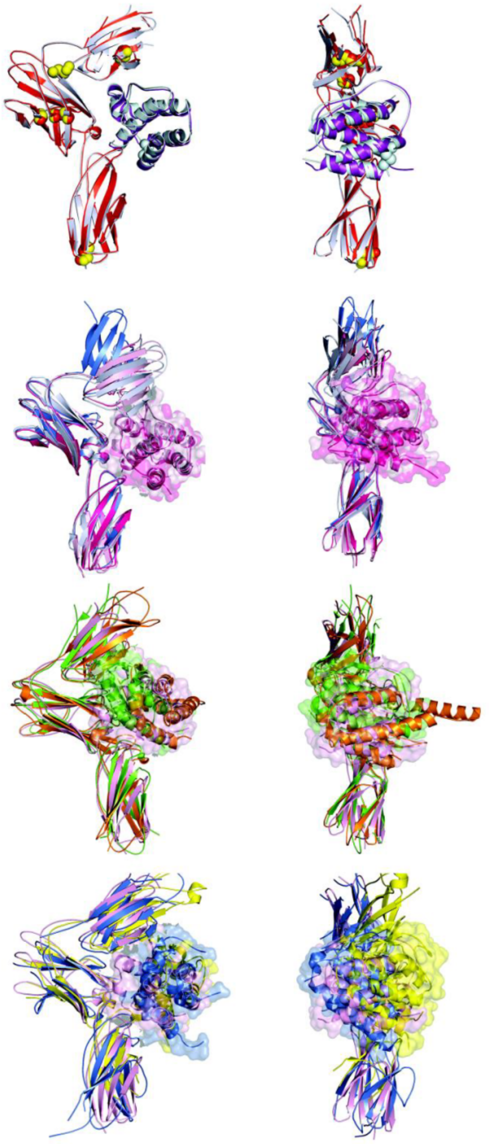

Supplementary Fig. 2. Comparison of wild-type and IL-3 K116W binary complexes with related cytokine binary (GM-CSF, IL-5 and IL-13) or ternary (IL-13) complexes. **a** Overlay of the two copies of the wild-type IL-3 binary complex observed in the crystallographic asymmetric unit via the α-subunit CRM (i.e. D2-D3). Copy one: IL3Rα coloured blue-grey and IL-3 purple. Copy two: IL3Rα coloured red and IL-3 light cyan. **b** Overlay of the IL3Rα open conformation (blue) and closed conformation (blue grey) (PDB ID: 4JZJ[1]) via the CRM with the wild-type IL-3 binary complex (light pink) and the IL-3 K116W binary complex (hot pink). **c** Overlay of GM-CSF (green), wild-type IL-3 (light pink) and IL-5 (orange) binary complexes via the CRM. **d** Overlay of the wild-type IL-3 binary complex (light pink), IL-13:IL13Rα1 component extracted from the

ternary complex (blue) and IL-13:IL13Rα2 binary complex (yellow) via the CRM. Side and front views shown for panels **a** - **d**. In panels **b** – **d**, a transparent molecular surface for the cytokines is also displayed. In panel **c**, only the molecular surface for the IL-5 monomer directly bound to IL5Rα is shown.

a

b

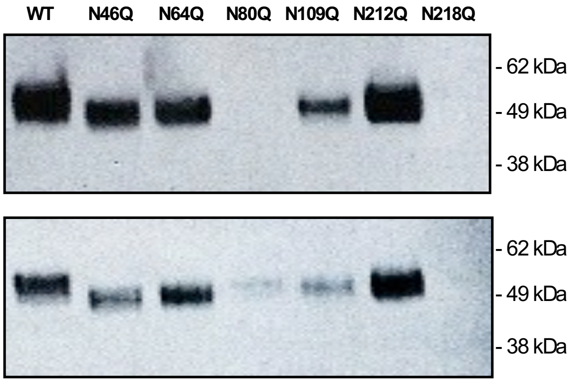

c

		Wild-type IL-3		
Cell	Receptor	n	K_D (nM)	*p*
COS	IL3Rα	8	140.6 ±16.9	-
COS	IL3Rα N212Q	3	157.7 ±33.8	0.59

d

	Wild-type IL-3		
IL3Rα	n	ED_{50} (ng ml^{-1})	*p*
Wild-type	6	0.29 ±0.11	-
N212Q	4	0.27 ±0.02	0.86

Supplementary Fig. 3. Critical role of selected N-linked glycosylation sites in IL3Rα protein expression but no critical role revealed for N212 by the IL3Rα N212Q mutation in IL-3 binding or signalling. Wild-type sIL3Rα-6xHis or mutants of each of the six predicted N-linked glycosylation sites, were expressed using transfected Freestyle™293 cells and supernatants immunoblotted using specific antibodies for IL3Rα **a** or 6xHis **b**. Soluble IL3Rα variants include wild-type (WT), N46Q, N64Q, N80Q, N109Q, N212Q and N218Q. Blots shown are representative of two independent experiments. **c** Binding of wild-type IL-3 to COS cells transfected with a plasmid encoding the N212Q form of IL3Rα was measured in saturation binding assays using radioiodinated cytokine as described[2,3]. Binding data for COS cells

expressing wild-type IL3Rα is taken from Table 2. Errors represent SEM from the indicated number (n) of experiments. Statistical significance of differences in binding between wild-type IL3Rα and N212Q form of IL3Rα (*p*) was determined using a 2-tailed unpaired t-test. **d** Functional activity of the IL3Rα N212Q mutant was measured in wild-type IL-3 dose-response proliferation assays using FDH cells transduced to co-express the βc subunit with either wild-type or N212Q forms of IL3Rα. Half-maximal responses (ED_{50}) were calculated from each experiment and averaged. Statistical significance of differences in functional response between wild-type IL3Rα and the N212Q forms of IL3Rα (*p*) was determined using a 2-tailed paired t-test.

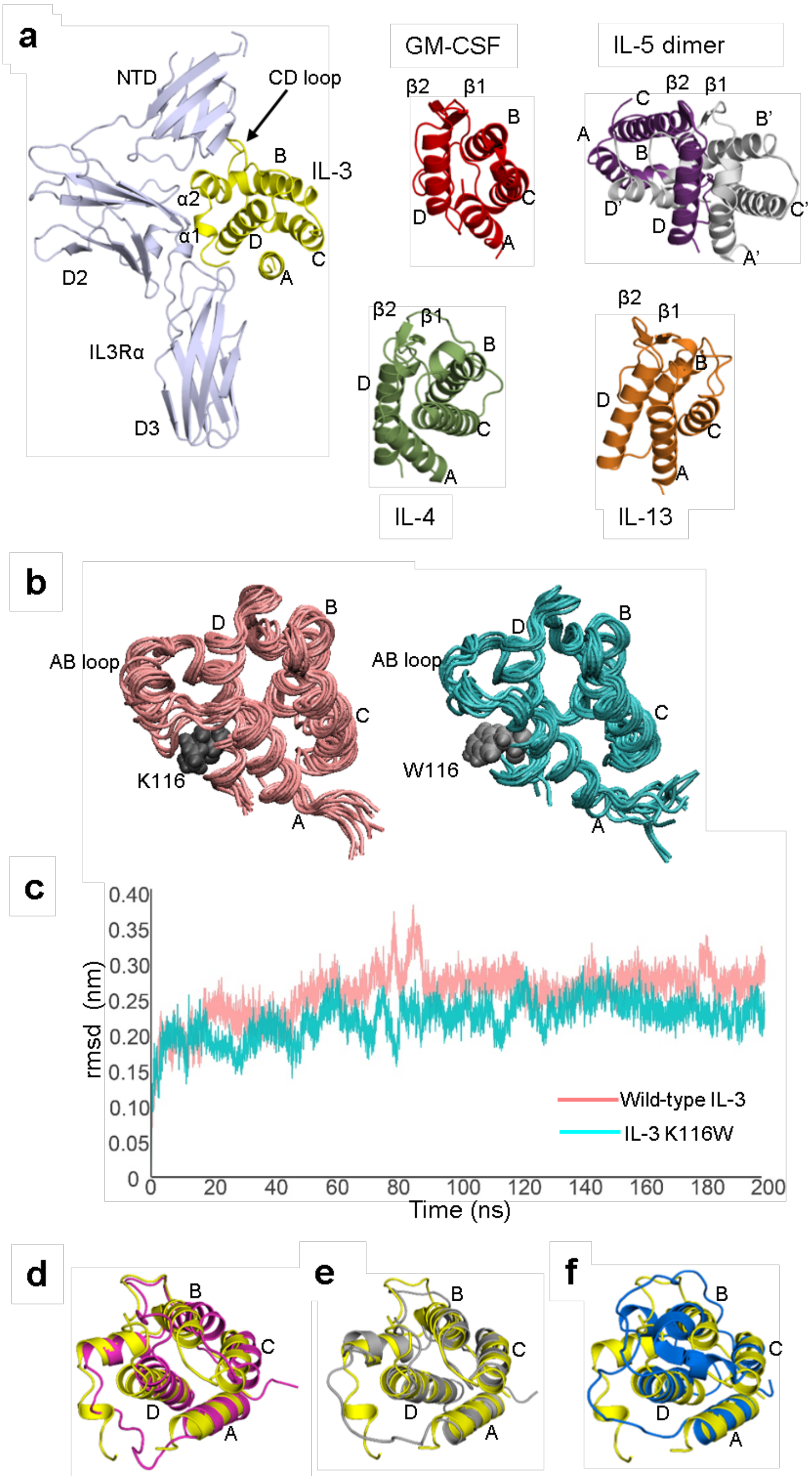

Supplementary Fig. 4. Comparison of IL-3, GM-CSF, IL-4, IL-5 and IL-13 cytokine structures.

a Wild-type IL-3 binary complex (IL-3 coloured yellow). IL-3 interacts with the IL3Rα NTD D-

strand via the α2-helix and the unstructured CD loop. GM-CSF, IL-5 homodimer, IL-4 and IL-13, respectively, extracted from their corresponding binary or ternary complexes after alignment of their cytokine-specific α-subunit with the IL3Rα CRM. The GM-CSF, IL-5 homodimer, IL-4 and IL-13 cytokines all interact with their respective α-subunit's NTD D-strand via main-chain interactions with the cytokine β2-strand. **b** Snapshots (every 20 ns) from the 200 ns all-atom solvent-explicit molecular dynamics (MD) simulation of wild-type (pink) and K116W (blue) human IL-3 (alone) showing that the IL-3 K116W superkine is less conformationally mobile. The locations of K116 and K116W on the AB loop are indicated by the grey spheres. **c** The rmsd of the IL-3 Cα atoms, over the course of the 200 ns MD simulations, indicates that wild-type IL-3 (alone) is more conformationally mobile than IL-3 K116W (alone). Colour scheme as in panel **b**. **d** Overlay of wild-type (yellow) and K116W (dark pink) human IL-3 extracted from the binary complex crystal structures. **e** Overlay of the wild-type human IL-3 crystal structure (yellow) with the solution NMR structure of the human IL-3 (grey; PDB ID: 1JLI,[4]). **f** Overlay of the wild-type human IL-3 crystal structure (yellow) with the solution NMR structure of the murine IL-3 (blue; PDB ID: 2L3O[5]).

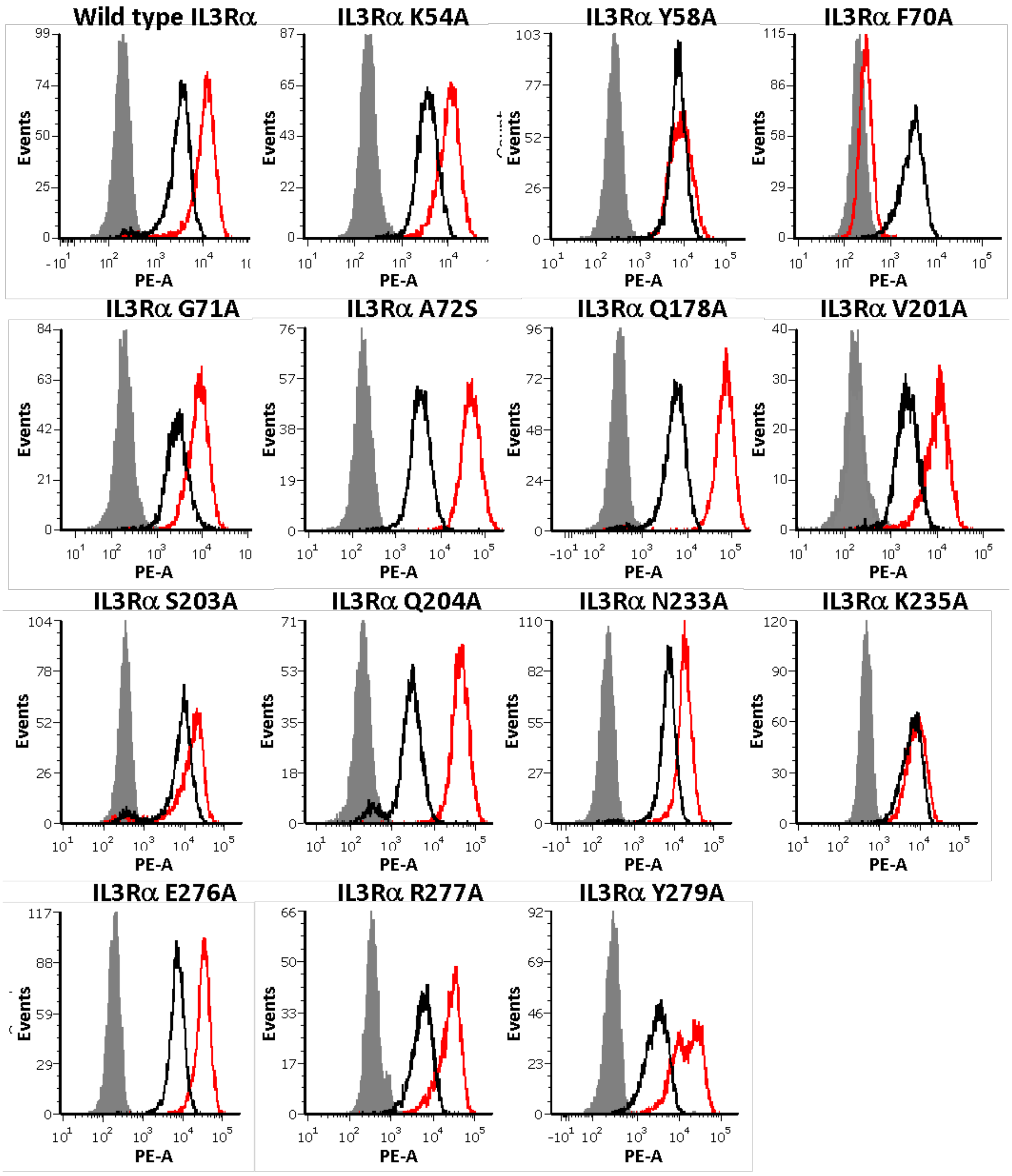

Supplementary Fig. 5. Expression of IL3Rα site 1 mutants. CTLL-2 cell lines stably expressing βc and wild-type or site 1 mutants forms of IL3Rα were stained with MAb's specific for IL3Rα (9F5), βc (1C1) or an isotype control (1B5) and cell surface expression determined by flow cytometry. Histograms show IL3Rα (red) and βc (black) expression with isotype control (filled grey).

a

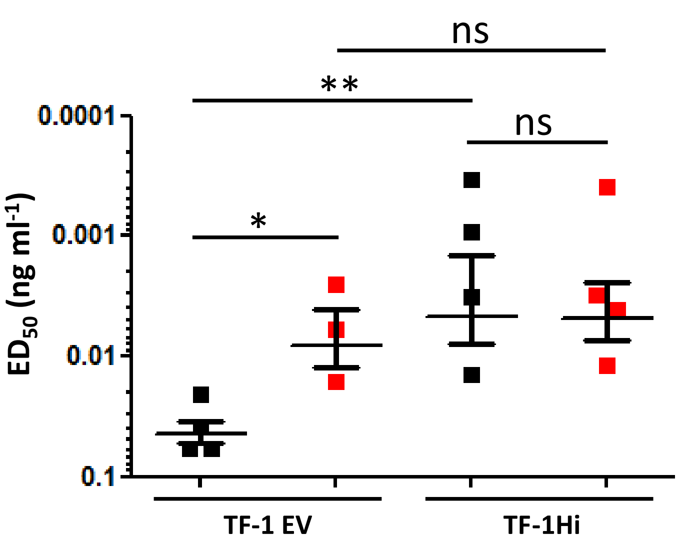

Cells	IL-3 n	IL-3 ED$_{50}$ (ng ml^{-1})	IL-3 *p*	IL-3 K116W n	IL-3 K116W ED$_{50}$ (ng ml^{-1})	IL-3 K116W *p*	P
TF-1 EV	4	0.044 ±0.009	-	3	0.008 ±0.0041	-	0.024
TF-1Hi	4	0.005 ±0.003	0.006	4	0.005 ±0.0025	0.504	0.947

b

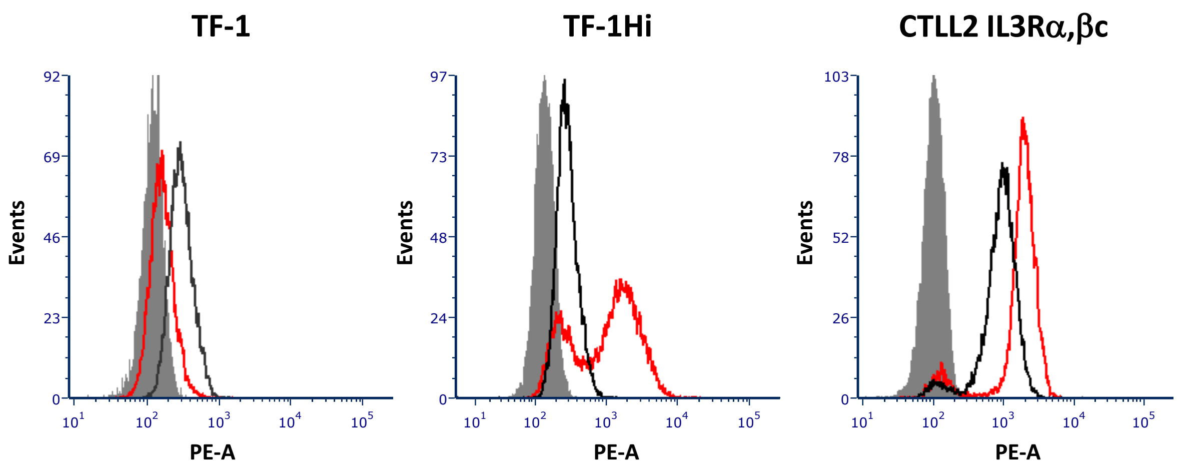

Supplementary Fig. 6. IL-3 function on TF-1 and TF-1Hi cells and IL-3 receptor expression on TF-1, TF-1Hi and the CTLL-2 cell line. **a** TF-1 cells transduced with a lentivirus encoding wild-type human IL3Rα (TF-1Hi) or empty vector (TF-1 EV), were stimulated with titrations of wild-type IL-3 (■) or IL-3 K116W (■). Half-maximal responses (ED$_{50}$) were calculated from each experiment and averaged. Errors represent SEM from the indicated number (n) of experiments. Statistical significance of differences in functional response between TF-1 EV and TF-1Hi (*p*) or

between wild-type IL-3 and IL-3 K116W (P) was determined using a 2-tailed unpaired t-test. ns $p > 0.05$, * $p < 0.05$, ** $p < 0.01$. **b** TF-1 cells expressing the endogenous βc and IL3Rα or TF-1Hi cells expressing endogenous βc with overexpressed IL3Rα, were stained with MAb's specific for IL3Rα (9F5), βc (1C1) or an isotype control (1B5) and cell surface expression determined by flow cytometry. CTLL-2 cell lines stably expressing βc and wild-type IL3Rα were used as a reference. Histograms show IL3Rα (red) and βc (black) expression with isotype control (filled grey).

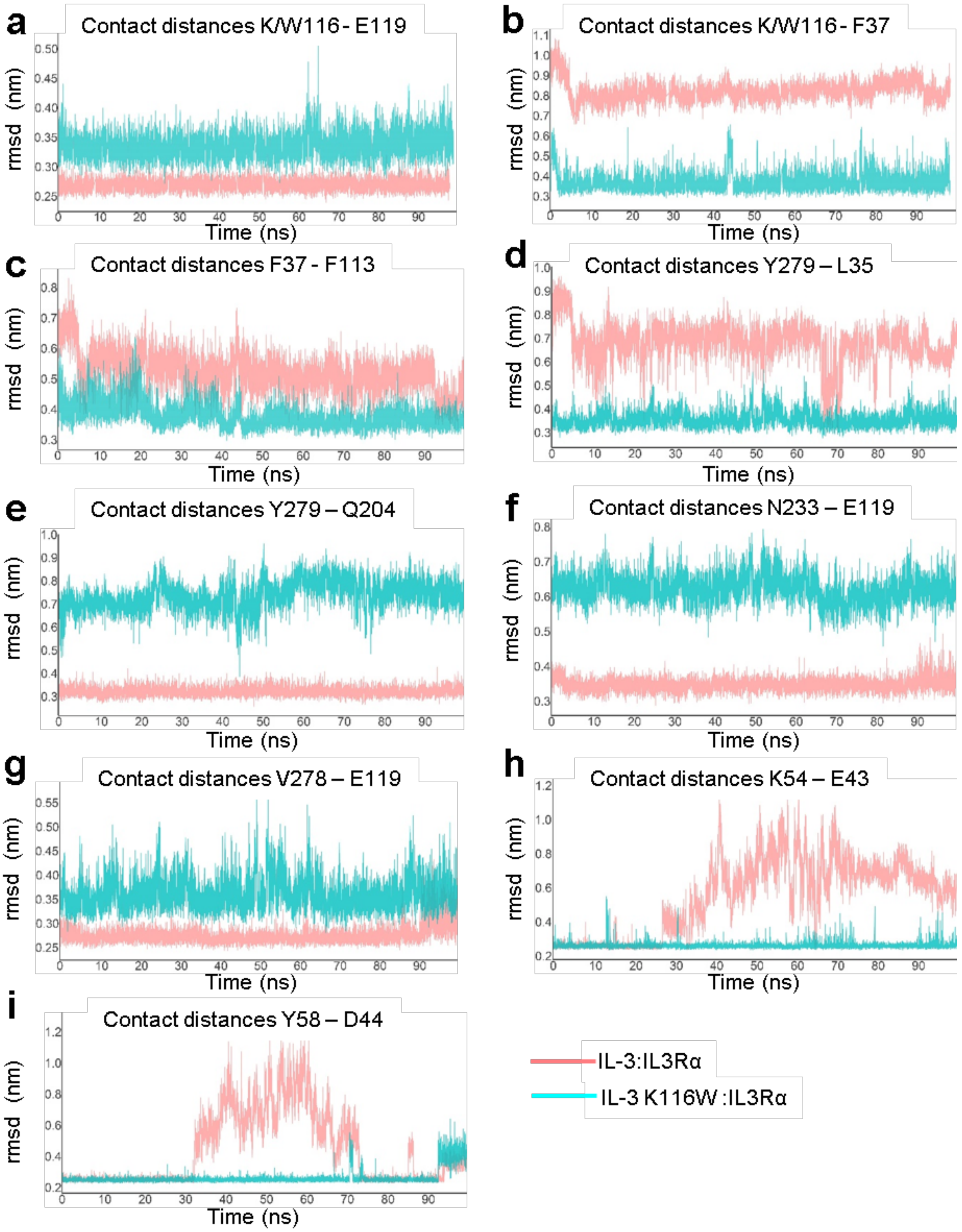

Supplementary Fig. 7. Contact distances between residue pairs during the 100 ns MD simulations of the wild-type and IL-3 K116W binary complexes. **a** The tryptophan side-chain of IL-3 K116W (blue line) reorientates so that it binds within a largely aromatic and hydrophobic

pocket defined by F37, T112, F113, T117, E119 and N120 of IL-3 and S203, Q204, N233, E276, V278 and Y279 of IL3Rα. The W116 reorientation results in the tryptophan being further away from IL-3 E119 than K116 in the wild-type (pink line) cytokine. **b** – **d** Unfurling of the α1-helix on the AB loop of IL-3 K116W enables the formation of the π-π interaction network between F37-K116W-F113. The conformational change in the AB loop is reflected by the shorter distance between IL-3 W116 and IL-3 F37 when compared to the K116 - F37 distance in the wild-type cytokine **b**, a shorter distance between IL-3 F37- IL-3 F113 in the IL-3 K116W binary complex **c** and a larger distance in the wild-type binary complex between IL-3 L35 – IL3Rα Y279 **d**. **e** – **g** The size of the site 1a pocket that W116 is located in (defined in panel **a** legend) is much larger than the corresponding K116 binding pocket, as reflected in the greater contact distances between the defined residue pairs IL3Rα Q204 - IL3Rα Y279 (**e**), IL-3 E119 - IL3Rα N233 (**f**) and IL-3 E119 - IL3Rα V278 **g**. **h** – **i** The reduced mobility of the IL3Rα NTD in the IL-3 K116W binary complex is reflected by the smaller variation in contact distance between residue pairs IL-3 E43 - IL3Rα K54 (**h**) and IL-3 D44 - IL3Rα Y58 **i**.

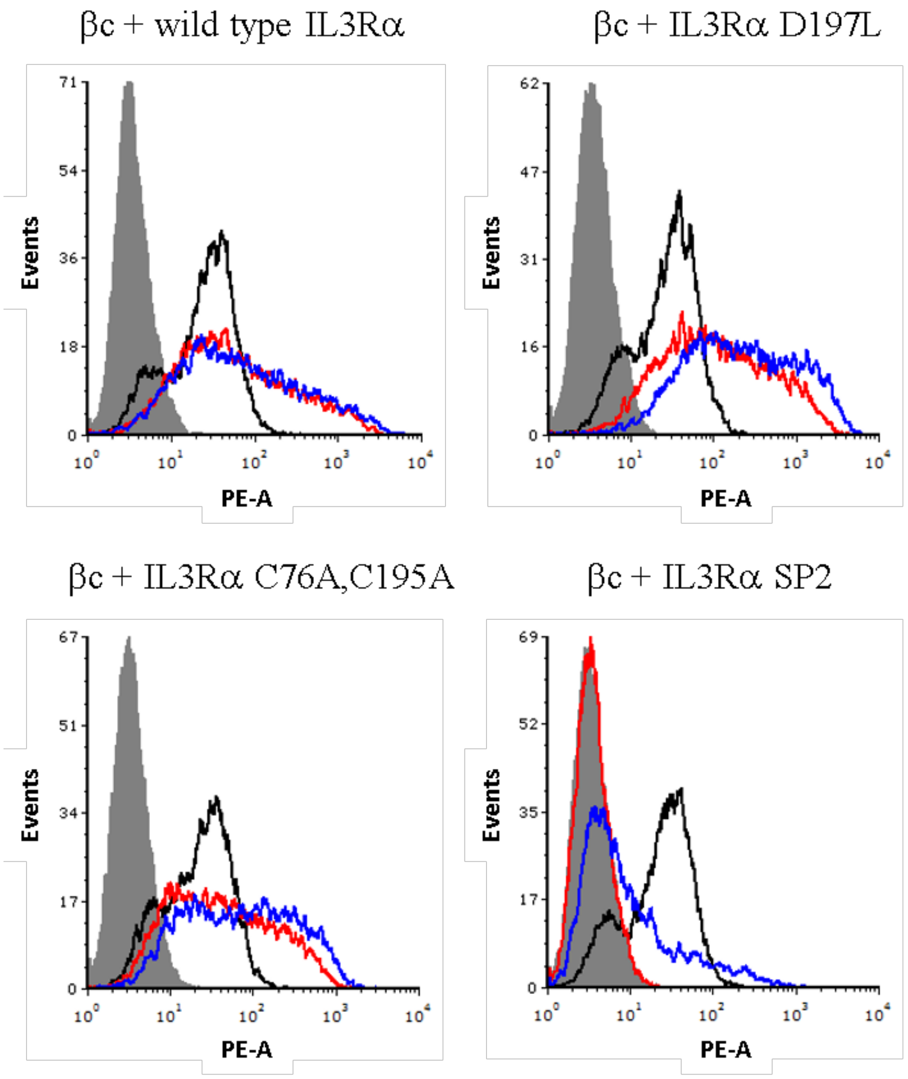

Supplementary Fig. 8. Expression of IL3Rα and βc on transfected HEK293-T-βc cells. HEK293-T cells stably expressing βc were transfected with plasmids encoding wild-type, D197L or C76A,C195A forms of full-length IL3Rα or IL3Rα SP2. Cells were stained with MAb's specific for IL3Rα (9F5, MAB301), βc (1C1) or an isotype control (1B5) and cell surface expression determined by flow cytometry. MAb 9F5 binds IL3Rα NTD and MAB301 binds IL3Rα D2-D3. Histograms show IL3Rα NTD (red), IL3Rα D2-D3 (blue) and βc (black) expression with isotype control (filled grey).

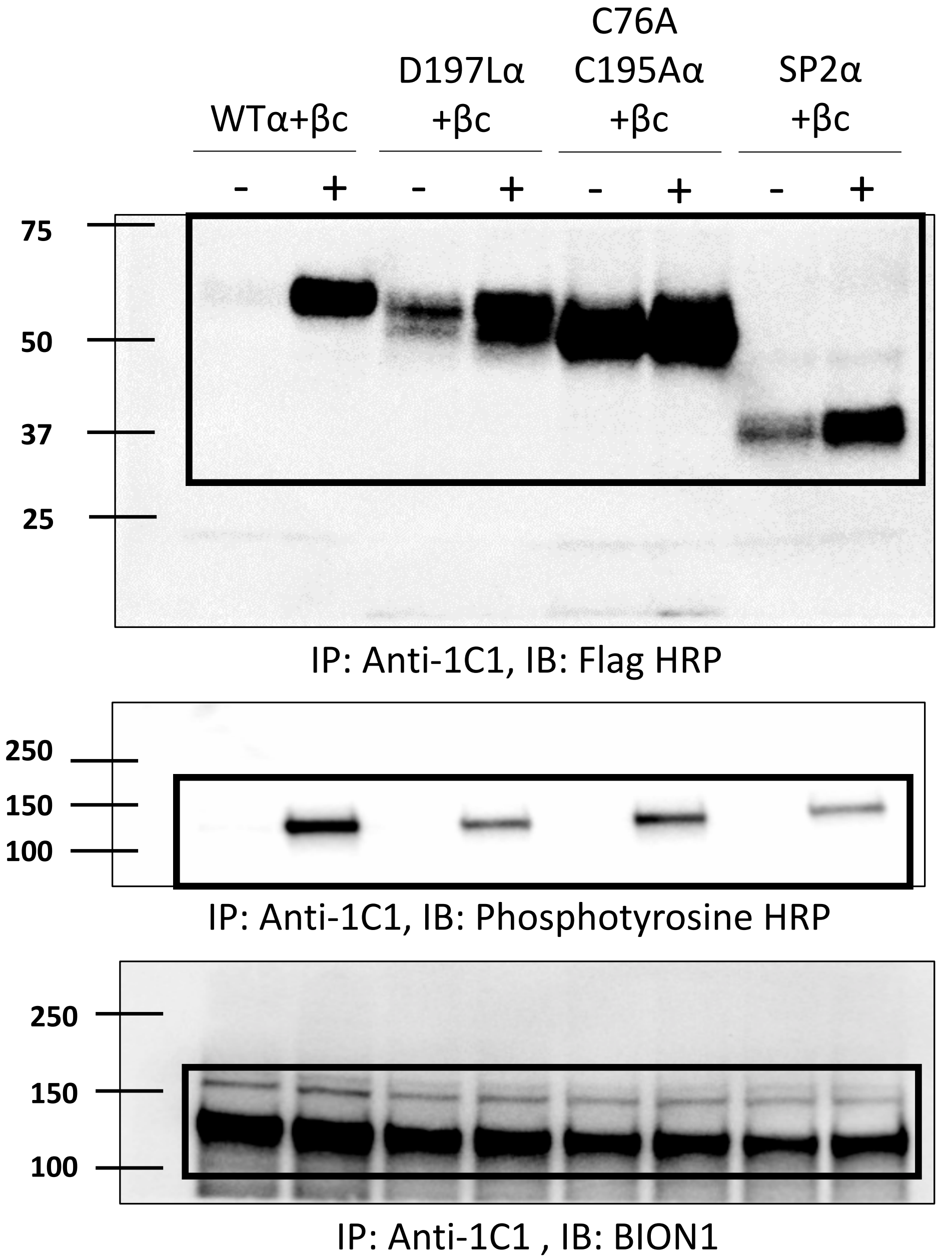

Supplementary Fig. 9. Cell lysates from HEK293T-βc cells transfected with wild-type (WT), D197L, C76A,C195A or SP2 forms of Flag-tagged IL3Rα and stimulated with 1.5 μg ml^{-1} IL-3 were immunoprecipitated (IP) with the βc antibody 1C1 conjugated Sepharose. Immunoprecipitates were immunoblotted (IB) with antibodies for IL3Rα (Flag HRP), phosphotyrosine (phosphotyrosine HRP) or total βc (BION1). Image shows the uncropped scan of all three immunoblots showing the position of molecular weight markers. The area within the thick lined box is the area included in Fig. 6f.

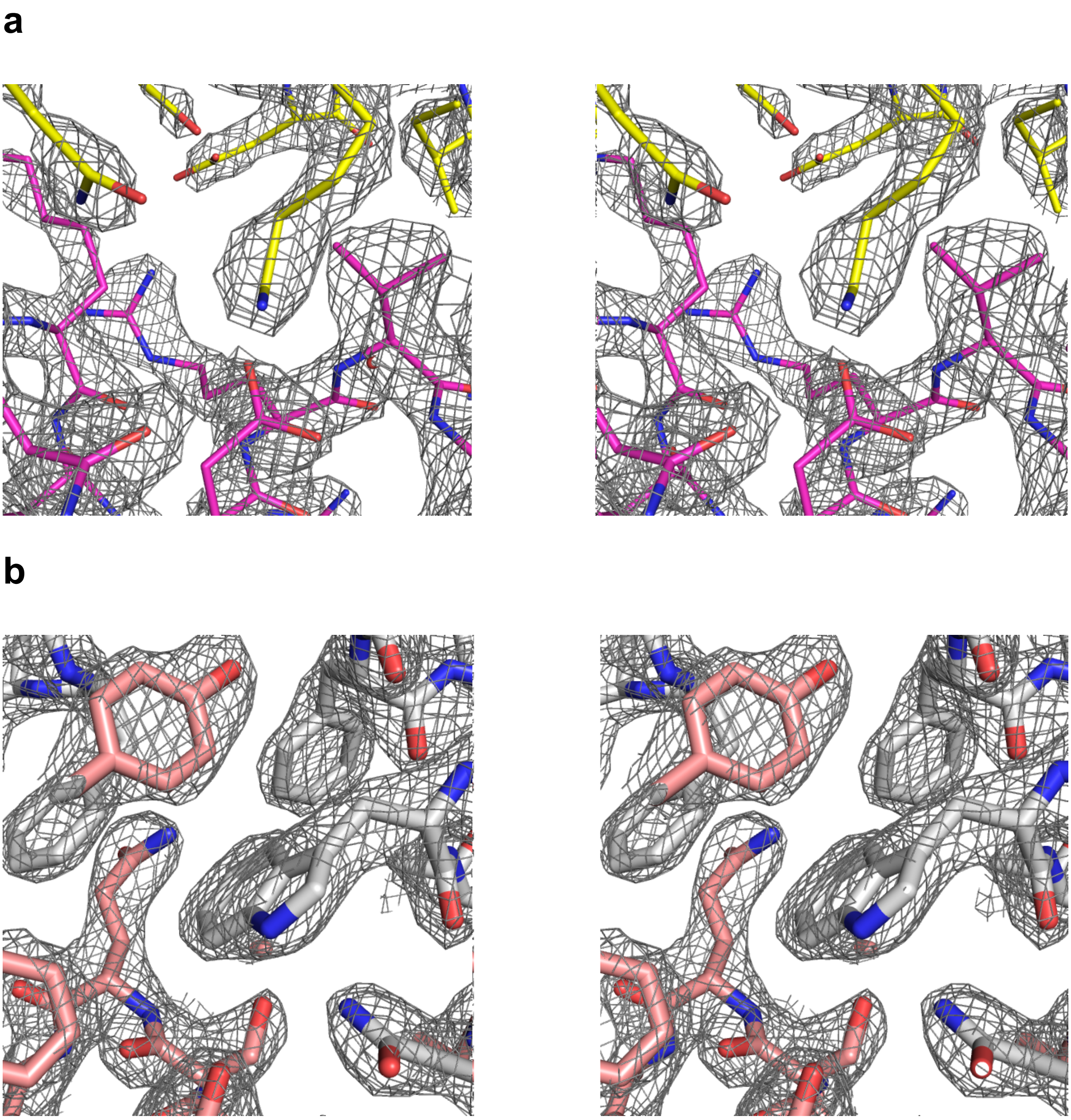

Supplementary Fig. 10. Stereo images of wild-type IL-3 and IL-3 K116W bound to IL3Rα. **a.** Stereo image of a portion of the electron density of wild-type IL-3 (yellow sticks) bound to IL3Rα (magenta sticks), centred on K116 of IL-3. The $2F_o$-F_c map is displayed, contoured to 1σ. **b.** Stereo image of a portion of the electron density of IL-3 K116W (grey sticks) bound to IL3Rα (salmon sticks), centred on W116 of the IL-3 mutant. The $2F_o$-F_c map is displayed, contoured to 1σ.

SUPPLEMENTARY TABLES

Supplementary Table 1 IL-3:IL3Rα contacts in the wild-type and K116W binary complexes

IL3Rα	Wild-type IL-3	IL-3 K116W
**NTD**		
K54	E43••	E43••
Y58	N41, E43, D44•, I47	D44•, E43, I47
P61	H95	H95
A62	H95•	H95•
Y67	T93	-
Q69	R94	-
F70	E43	E43
G71	E43•, D46	E43•, D46
A72	G42, E43•	E43•
**D2**		
Q178	T117•, N120, A121	T117•, N120, A121
S179	M49	M49
V201	Q45	Q45, M49
S203	K116•, N120•	W116, N120
Q204	F37, F113, K116•	F37, Q45•, F113, W116
**D3**		
F232	N120	N120
N233	K116•, N120	N120•, W116
R234	N120•, Q122	E119, N120•, A123
K235	S17, E119	E119••, S17•
R255	D21••, S76, I77	D21••
E276	K116	W116
R277	D21••, K28•	D21••, K28•
V278	T112, K28•	K28•, T112, W116
Y279	F37, T112, F113, K116	L35, F37, T112, F113, W116
E280	K28••	K28••
F281	F37	-

•Hydrogen bond, ••Salt bridge. Interactions listed are within a 3.9-Å distance cutoff.

Supplementary Table 2 Key residues in IL3Rα site 1a and site 1b important for IL-3 signalling

	IL-3			IL-3 K116W			
IL3Rα	n	ED_{50} (ng ml^{-1})	*p*	n	ED_{50} (ng ml^{-1})	*p*	P
Wild-type	10	0.028 ±0.007	-	10	0.018 ±0.005	-	0.25
K54A	6	0.26 ±0.04	<0.0001	6	0.072 ±0.025	0.016	0.002
Y58A	6	0.24 ±0.07	0.002	5	0.027 ±0.008	0.29	0.028
F70A	5	46.6 ±5.6	<0.0001	5	18.8 ±8.5	0.006	0.026
G71A	5	0.18 ±0.04	0.0002	4	0.047 ±0.023	0.093	0.033
A72S	3	0.24 ±0.11	0.0025	3	0.16 ±0.06	0.0009	0.54
Q178A	5	0.13 ±0.05	0.012	4	0.14 ±0.07	0.016	0.88
V201A	4	0.24 ±0.06	0.0001	4	0.06 ±0.03	0.031	0.036
S203A	3	0.057 ±0.023	0.12	3	0.023 ±0.009	0.64	0.23
Q204A	3	0.26 ±0.14	0.007	3	0.29 ±0.12	0.0007	0.89
N233A	4	0.49 ±0.13	<0.0001	4	0.029 ±0.007	0.24	0.014
K235A	6	0.04 ±0.01	0.32	5	0.009 ±0.006	0.22	0.035
E276A	5	0.27 ±0.08	0.0008	5	0.044 ±0.008	0.0097	0.024
R277A	5	0.042 ±0.017	0.38	5	0.072 ±0.041	0.080	0.52
Y279A	6	0.88 ±0.34	0.005	6	0.074 ±0.044	0.12	0.039

Functional activity of IL3Rα mutants was measured in IL-3 or IL-3 K116W dose-response proliferation assays using CTLL-2 cell lines transduced to co-express the βc subunit with either wild-type or mutant forms of IL3Rα. IL3Rα residues are numbered according to the structure determined using the K144NR splice-variant (**Supplementary Fig. 1c**). Because these functional studies used the K144 splice-variant, residues beyond 144 are labelled +1 to match the structural data. Half-maximal responses (ED_{50}) were calculated from each experiment and averaged. Errors represent standard error of the mean (SEM) from the indicated number (n) of experiments. Statistical significance of differences in functional response between wild-type IL3Rα and the mutant forms of IL3Rα (*p*) or between IL-3 and IL-3 K116W (P) was determined using a 2-tailed unpaired t-test.

Supplementary Note 1

Interaction of wild-type IL-3 with IL3Rα. Multiple IL-3 residues interact at site 1a and site 1b in the IL-3:IL3Rα binary complex structure. Residues S17, D21, Q45, F113, K116 and Q122 in IL-3 interact through site 1a and are known to be functionally important[6-9]. IL-3 residues F37, F113, K116 and N120 interact with IL3Rα residues in the D2-D3 linker region; residues S17, K116, E119, N120 and Q122 interact with residues in the IL3Rα BC loop of D3; residue D21 forms a salt bridge with both R255 in the DE loop of IL3Rα D3 and R277 in the FG loop of D3. In addition, IL-3 T112, F113 and K116 interact with the IL3Rα FG loop of D3 (**Fig. 1c,d**). IL-3 residues E43, D44 and D46 interact exclusively through site 1b[8]. IL-3 residue E43 forms a salt bridge with K54 in the C-strand of the IL3Rα NTD, hydrogen bonds to the main-chain amides of G71 and A72 in the EF loop of the NTD and forms non-polar contacts with Y58 in the D-strand of the IL3Rα NTD, a residue which also hydrogen bonds to IL-3 residue D44. Residue D46 can potentially interact with the main-chain carbonyl of G71 in the FG loop of the IL3Rα NTD (**Fig. 1b**). Interestingly, two IL-3 residues that form hydrogen bonds with IL3Rα residues, IL-3 K28 forms a salt bridge with E280 in the FG loop of IL3Rα D3 and IL-3 E119 bonds with K235 in the BC loop of IL3Rα D3 (**Fig. 1d**), appear to be tolerant to substitution[10] as are the interacting residues in IL3Rα[1].

Comparison of the two receptor complexes in the wild-type IL3:IL3Rα crystal structure. Two complexes were found in the asymmetric unit, designated by their chain identifiers AB and GI, and they overlay closely with root-mean-square deviation (rmsd) on Cα atoms of 0.7-Å (**Supplementary Fig. 2a**). Despite the overall similarity of the two complexes there are a few subtle differences. In complex GI, the amine side-chain of K116 makes hydrogen bonds to the side-chains of S203 and Q204 in the D2-D3 linker and to the amide side-chain of N233 in the D3 domain of IL3Rα. In contrast, in complex AB the K116 amine group points away from Q204,

instead forming a salt bridge with E276 as well as the hydrogen bond to N233 in the D3 domain. Also in complex GI, the carboxylate group of D21 in IL-3 makes an additional salt bridge with the guanidino moiety of the flexible side-chain of R255 from the D-strand of D3 IL3Rα.

Comparison of the bound IL-3 structures with free IL-3. Although this is the first crystal structure of human IL-3, there are NMR structures of a heavily mutated human IL-3 (PDB ID: 1JLI)[4] and a murine IL-3 with a single mutation (PDB ID: 2L3O)[5]. The structures overlay closely (rmsd over the Cα atoms = 1.3-Å) with essentially the same secondary structure, however the murine structure shows an extended conformation of the AB loop (**Supplementary Fig. 4d-f**).

The IL3Rα NTD position in the binary complex is an intermediary between the IL3Rα open and closed structures. We have previously solved the structure of IL3Rα bound to an antibody Fab revealing two differing placements ("open" and "closed") of the NTD relative to the CRM[1]. This suggested mobility within the receptor that is likely due to a long flexible linker attaching the NTD to the CRM. The receptor from the binary complexes overlays closely via the CRM (< 1-Å Cα rmsd) to the open and closed conformations of IL3Rα seen in the Fab complex (**Supplementary Fig. 2b**). A side-on view shows the position of the NTD lies between the positions of the open and closed IL3Rα (**Supplementary Fig. 2b**). There are no major differences in side-chain conformations in the CRM and in the NTD-D2 interface.

IL3Rα contains N-linked glycans that are required for expression. Electron density representing glycan moieties was observed at glycosylation sites N80, N109 and N218 in IL3Rα. We previously reported that N80 glycosylation is important for IL3Rα expression[1], as is glycosylation at N218 and to a lesser extent N109 (**Supplementary Fig. 3a,b**).

While we previously reported that N212 contains no N-linked glycans[1], we observed that the sIL3Rα N212Q variant was expressed at a higher level than wild-type sIL3Rα (T.R.H. unpublished observation) so we used this variant, known as sIL3Rα ΔN5, for our structural studies. The location of N212 in the IL3Rα structure suggests that conservative mutations of this residue are unlikely to affect IL3Rα function (**Fig. 1a**) and we have confirmed this by assessing the impact of the IL3Rα N212Q mutation on IL-3 binding and function. The binding of IL-3 to COS cells expressing IL3Rα N212Q was indistinguishable from the binding to COS cells expressing wild-type IL3Rα (K_D = 157.7 nM vs 140.6 nM respectively, **Supplementary Fig. 3c**). FDH cells co-expressing βc, and wild-type IL3Rα or IL3Rα N212Q proliferated in response to wild-type IL-3 stimulation with comparable potency (IL-3 ED_{50} = 0.29 ng ml^{-1} and 0.27 ng ml^{-1}, respectively, **Supplementary Fig. 3d**).

Structural comparison of cytokine structures. Prior to the crystallisation of the wild-type IL-3 and K116W IL-3 binary complexes in this study, only the NMR structures of a heavily mutated human IL-3 (PDB ID: 1JLI[4]) and a singly mutated murine IL-3 (PDB ID: 2L3O[5]) were available. The top ranked NMR models for the mutated human (rmsd = 1.2-Å) and murine (rmsd = 2.3-Å) IL-3 were aligned via the Cα of the four α-helices with the human wild-type and K116W cytokines in the IL-3 binary complex crystal structures (**Supplementary Fig. 4d-f**). In the two copies of the wild-type IL-3 binary complex in the asymmetric unit, density for residues 31 and 32 (chain B) and residue 32 (chain I) on the AB loop is not visible. The locations of α-helices A, B, C, D, α1 and α2, and the C16-C84 disulfide bond, for the wild-type IL-3 are indicated in **Fig. 1a** and **Supplementary Fig. 1c**. In the IL-3 K116W binary complex, the density for the AB loop is complete and the single-turn α1-helix is partially unfolded to facilitate the formation of the π-π interaction network between F37-K116W-F113 (**Fig. 4**, **Supplementary Figs. 1c, 4b**). Although the two binary complexes are structurally similar (rmsd Cα = 0.5-Å), cytokine α-helices

B, C and D in the IL-3 K116W binary complex are shifted slightly away from the α-subunit (i.e. shifted to the right when viewed as in **Supplementary Fig. 4d**) compared to the wild-type IL-3 location. Helices A-D of the top ranked mutated human IL-3 NMR model align well with those in the wild-type IL-3 binary complex (**Supplementary Fig. 4e**); however, the two-turn α2-helix in the AB loop of the NMR structure is slightly closer to the IL3Rα NTD. In addition, the AB loop prior to the α2-helix adopts a significantly different conformation in the NMR structure. Although helices A-D of the top ranked murine IL-3 NMR model align well with those for the human IL-3 binary complex (**Supplementary Fig. 4f**), the AB, BC, CD loops adopt very different conformations. In particular, the two-turn α2-helix of the human IL-3 AB loop corresponds to a single-turn α1-helix in the murine IL-3 (**Supplementary Fig. 4d,f**).

Structural comparison of the IL-3 binary complex and related cytokine receptor structures. The "wrench-like" conformation adopted by IL3Rα in binding IL-3 is reminiscent of conformations observed for IL-5, GM-CSF and IL-13 receptors. The interface between the α-subunit and cytokine is extensive and similar for each binary complex with a total buried surface area of ~1,136-$Å^2$ for IL-3:IL3Rα compared to ~1,115-$Å^2$ for IL-5:IL5Rα and ~1,175-$Å^2$ for GM-CSF:GMRα. The IL-3:IL3Rα complex interface has a similar shape complementarity (S_c)[11] value of 0.69 compared to 0.64 for IL-5:IL5Rα and 0.66 for GM-CSF:GMRα. IL3Rα also superimposes reasonably well with IL5Rα and GMRα despite low shared sequence identity (18-32%), with the CRMs particularly similar (rmsd over the Cα atoms of 1.8-Å and 2.3-Å, respectively) and the D2-D3 elbow angles being the same. The greatest difference between the GM-CSF, IL-5 and IL-3 α-subunits is the positioning of the NTD relative to the CRM (**Supplementary Fig. 2**).

The βc cytokine receptor family are structurally related to the IL-13 receptor family[12]. Crystal structures of the IL13Rα1 and IL13Rα2 receptors reveal they also have a three FnIII

domain architecture that forms a "wrench-like" conformation around IL-13 (PDB: 3BPO, 3LB6)[13,14] or IL-4 in the Type II ternary complex (PDB: 3BPN)[13] (**Supplementary Fig. 2d**). The overall secondary structure in the CRMs of the IL3Rα and IL13Rα1/2 receptor subunits is conserved; however, there is significant variation in the angle and placement of the β-sheets and loops (rmsd over the Cα atoms of 4.8-Å and 3.9-Å, respectively). The placement of the NTD relative to the CRM is another significant difference between the complexes (**Fig. 2, Supplementary Fig. 2**).

The wild-type IL-3 and IL-3 K116W binary complexes were also aligned via the IL3Rα CRM with the GM-CSF binary complex (rmsd Cα = 2.3-Å; PDB ID: 4RS1[15]), the IL-5 binary complex (rmsd Cα = 1.8- Å; PDB ID: 3QT2[16]), with IL13Rα1 in the IL-4 ternary complex (rmsd Cα = 5.2-Å; PDB ID: 3BPN[13]), with IL13Rα1 in the IL-13 ternary complex (rmsd Cα = 4.8-Å; PDB ID: 3BPO[13]), and IL13Rα2 in the binary complex (rmsd Cα = 2.8-Å; PDB ID: 3LB6[14]) to illustrate the unique way IL-3 interacts with the α-subunit NTD (**Supplementary Figs. 2c-d, 4a**). GM-CSF, IL-4, IL-5 and IL-13 all contain two short antiparallel β-strands, β1 located in the AB loop and β2 in the CD loop. The cytokine β2-strand interacts via several main-chain:main-chain hydrogen bonds with the D-strand of the α-subunit NTD thereby extending the NTD β-sheet formed by the C-, D-, F- and G-strands. IL-3 does not contain these two short antiparallel β-strands and instead interacts with the IL3Rα NTD via residues located on the α2-helix and the unstructured CD loop.

SUPPLEMENTARY REFERENCES

1. Broughton, S.E. et al. Dual mechanism of interleukin-3 receptor blockade by an anti-cancer antibody. *Cell Rep* **8**, 410-9 (2014).
2. Woodcock, J.M., Bagley, C.J., Zacharakis, B. & Lopez, A.F. A single tyrosine residue in the membrane-proximal domain of the granulocyte-macrophage colony-stimulating factor, interleukin (IL)-3, and IL-5 receptor common beta-chain is necessary and sufficient for high affinity binding and signaling by all three ligands. *J Biol Chem* **271**, 25999-6006 (1996).
3. Woodcock, J.M. et al. Three residues in the common beta chain of the human GM-CSF, IL-3 and IL-5 receptors are essential for GM-CSF and IL-5 but not IL-3 high affinity binding and interact with Glu21 of GM-CSF. *EMBO J* **13**, 5176-85 (1994).
4. Feng, Y., Klein, B.K. & McWherter, C.A. Three-dimensional solution structure and backbone dynamics of a variant of human interleukin-3. *J Mol Biol* **259**, 524-41 (1996).
5. Yao, S., Young, I.G., Norton, R.S. & Murphy, J.M. Murine interleukin-3: structure, dynamics, and conformational heterogeneity in solution. *Biochemistry* **50**, 2464-77 (2011).
6. Bagley, C.J. et al. A discontinuous eight-amino acid epitope in human interleukin-3 binds the alpha-chain of its receptor. *J Biol Chem* **271**, 31922-8 (1996).
7. Barry, S.C. et al. Two contiguous residues in human interleukin-3, Asp21 and Glu22, selectively interact with the alpha- and beta-chains of its receptor and participate in function. *J Biol Chem* **269**, 8488-92 (1994).
8. Klein, B.K. et al. The receptor binding site of human interleukin-3 defined by mutagenesis and molecular modeling. *J Biol Chem* **272**, 22630-41 (1997).

9. Lopez, A.F. et al. Residue 21 of human granulocyte-macrophage colony-stimulating factor is critical for biological activity and for high but not low affinity binding. *EMBO J* **11**, 909-16 (1992).

10. Olins, P.O. et al. Saturation mutagenesis of human interleukin-3. *J Biol Chem* **270**, 23754-60 (1995).

11. Lawrence, M.C. & Colman, P.M. Shape complementarity at protein/protein interfaces. *J Mol Biol* **234**, 946-50 (1993).

12. Broughton, S.E. et al. The betac receptor family - Structural insights and their functional implications. *Cytokine* **74**, 247-58 (2015).

13. LaPorte, S.L. et al. Molecular and structural basis of cytokine receptor pleiotropy in the interleukin-4/13 system. *Cell* **132**, 259-72 (2008).

14. Lupardus, P.J., Birnbaum, M.E. & Garcia, K.C. Molecular basis for shared cytokine recognition revealed in the structure of an unusually high affinity complex between IL-13 and IL-13Ralpha2. *Structure* **18**, 332-42 (2010).

15. Broughton, S.E. et al. Conformational Changes in the GM-CSF Receptor Suggest a Molecular Mechanism for Affinity Conversion and Receptor Signaling. *Structure* **24**, 1271-81 (2016).

16. Patino, E. et al. Structure analysis of the IL-5 ligand-receptor complex reveals a wrench-like architecture for IL-5Ralpha. *Structure* **19**, 1864-75 (2011).
